# A novel bovine leukemia virus peptide vaccine targeting susceptible cattle-Estimating vaccine effectiveness using susceptible cattle constructed by fertilized ovum transplantation

**DOI:** 10.1186/1742-4690-12-S1-P50

**Published:** 2015-08-28

**Authors:** Shin-nosuke Takeshima, Lanlan Bai, Pan He, Yuki Matsumoto, Junko Kohara, Tsunao Hirai, Takashi Omori, Tetsuo Nunoya, Yusuke Yamamoto, Takeo Hidaka, Kyoko Togamura, Kazuhiro Matoba, Yoko Aida

**Affiliations:** 1 Viral Infectious Diseases Unit, RIKEN, Wako, Saitama, Japan; 2 Animal Research Center, Hokkaido Research Organization, Shintoku, Hokkaido, Japan; 3 Nippon Institute for Biological Science, Ome, Tokyo, Japan; 4 Hiroshima prefectural technology research institute, Hiroshima, Hiroshima, Japan; 5 North Hiroshima Livestock office, Shobara, Hiroshima, Japan; 6 National Institute of Livestock and Grassland Science, Nasushiobara, Tochigi, Japan

## 

Bovine leukemia virus (BLV), the aetiological agent of the enzootic bovine leukosis, was widely spread in the worldwide, and it is desired to develop vaccine. However, the disease progression is greatly affected by host factors, and the difference of disease susceptibility is caused the reason to make hard to estimate the BLV vaccine effect. In this study, we confirmed the association of the genotype of cattle major histocompatibility complex (BoLA) class II DRB3 gene and disease susceptibility by experimentally infection study using the cattle made by fertilized ovum transplantation technique. Susceptibility cattle were also used for estimating the effect of our developed new p12-4R1/gp51R1 peptide-conjugated Carbonate apatite (CO3Ap) vaccine and succeeded to detect the novel vaccine's effects such as A) pathologic suppression, B) Transmission interference, and C) infection inhibition. We produced six heads of disease susceptibility cattle (BoLA-DRB3*1601/*1601) which made by fertilized ovum transplantation technique, and also prepared four normal cattle (BoLA-DRB3*1501/*2703, DRB3*1501/*0503 and DRB3*1501/*1201). These cattle were attacked by BLV (copy number: 3-4 x 107), and measured the proviral load, lymphocyte count, and antibody titer for 161 days (susceptibility cattle) and 119 days (normal cattle) for estimating the disease susceptibility and also the vaccine effect. Within the three kind of test, A) pathologic suppression effect was most strong. In six BLV-infected susceptibility cattle, three vaccinated group were significantly suppressed the proliferation of proviral load, lymphocyte count, and CD5+B cell count, compared with unvaccinated group. On the contrary, in four normal cattle test, there is no significantly difference between the vaccinated group and control group because of high host factor's effect. This result clearly showed that the advantage of using susceptibility cattle for estimating the effect of anti-BLV vaccine, drugs or any treatments, and the BoLA genotyping is one of the good markers for using these strategies.

